# Can transcranial photobiomodulation improve cognitive function in TBI patients? A systematic review

**DOI:** 10.3389/fpsyg.2024.1378570

**Published:** 2024-06-17

**Authors:** Jia Zeng, Chen Wang, Yuan Chai, Danyun Lei, Qiuli Wang

**Affiliations:** ^1^School of Kinesiology and Health, Capital University of Physical Education and Sports, Beijing, China; ^2^Xinyang Central Hospital, Xinyang, China; ^3^Department of Physical Education, Xinyang University, Xinyang, China; ^4^Independent Researcher, Xinyang, Henan Province, China

**Keywords:** transcranial photobiomodulation, cognitive function, traumatic brain injury, brain function, parameters

## Abstract

**Introduction:**

Transcranial photobiomodulation (tPBM) is a non-invasive neuromodulation technology which has become a promising therapy for treating many brain diseases. Although it has been confirmed in studies targeting neurological diseases including Alzheimer’s and Parkinson’s that tPBM can improve cognitive function, the effectiveness of interventions targeting TBI patients remains to be determined. This systematic review examines the cognitive outcomes of clinical trials concerning tPBM in the treatment of traumatic brain injury (TBI).

**Methods:**

We conducted a systematic literature review, following the PRISMA guidelines. The PubMed, Web of Science, Scopus, EMBASE, and Cochrane Library databases were searched before October 31, 2023.

**Results:**

The initial search retrieved 131 articles, and a total of 6 studies were finally included for full text-analysis after applying inclusion and exclusion criteria.

**Conclusion:**

Results showed improvements in cognition for patients with chronic TBI after tPBM intervention. The mechanism may be that tPBM increases the volume of total cortical gray matter (GM), subcortical GM, and thalamic, improves cerebral blood flow (CBF), functional connectivity (FC), and cerebral oxygenation, improving brain function. However, due to the significant heterogeneity in application, we cannot summarize the optimal parameters for tPBM treatment of TBI. In addition, there is currently a lack of RCT studies in this field. Therefore, given this encouraging but uncertain finding, it is necessary to conduct randomized controlled clinical trials to further determine the role of tPBM in cognitive rehabilitation of TBI patients.

## 1 Introduction

TBI is a serious global public health issue that has attracted more and more attention from all sectors of society (Mondello et al., 2022). Studies have shown that the incidence rate of TBI is 939 per 100,000 people. Therefore, approximately 69 million people worldwide will suffer from TBI every year (Dewan et al., 2018). The disease affects about 2.5 million people every year just in America (Kwon et al., 2016).

Undoubtedly, TBI can lead to physical, cognitive, and behavioral impairments, all of which can lead to various limitations. In terms of cognitive function – attention, processing speed, language, long- and short-term memory, and executive function are most often affected (Zoccolotti et al., 2000; Vallat-Azouvi et al., 2007; Slovarp et al., 2012; Vakili and Langdon, 2016; Bosco et al., 2017; Shumskaya et al., 2017; Lee and Kim, 2018; Neville et al., 2019). These injuries are usually the main goals of rehabilitation plans after TBI (Nousia et al., 2022).

TBI is caused by external forces, often motor vehicle accidents, attacks, falls, sports injuries, or explosive injuries during military service (Menon et al., 2010; Hamblin, 2018). Some scholars believe these brain injuries may be caused by rotational (angular) force, linear (translational) forces, or the blunt force that decelerates during impact (Mckee and Daneshvar, 2015; Blennow et al., 2016). The cerebral cortex, including prefrontal cortex, is often injured in head trauma (Kato et al., 2007). Because the prefrontal cortex is an important brain area that controls cognitive function, TBI patients often suffer from cognitive dysfunction (Miller, 2000; Rabinowitz and Levin, 2014). The specific manifestations are that patients have abnormalities in attention, comprehension, processing speed, memory, language, thinking, judgment and learning abilities which seriously affect their work and life. Therefore, cognitive rehabilitation for TBI is crucial.

In fact, due to the complexity of brain structure, there is currently no recognized method for treating TBI, although some research methods are conducting tests in acute (neuroprotective) and chronic (neurorehabilitation) environments (Loane and Faden, 2010). But it cannot be denied that non-invasive technology has certain advantages in the treatment of brain diseases (Liew et al., 2014). In fact, transcranial electrical stimulation (TES) and transcranial magnetic stimulation (TMS) have been applied clinically and have achieved some therapeutic effects (Monai et al., 2016; Zhang et al., 2021). However, the therapeutic effects of these technologies are still limited and not without complications, such as epilepsy (Woollams et al., 2017). In addition, people are increasingly disappointed with drugs that treat brain function. Based on the above reasons, in recent years, people have become more and more interested in the application of tPBM in TBI (Naeser and Hamblin, 2011, 2015; Hamblin, 2016; Hennessy and Hamblin, 2016). tPBM has almost no adverse side effects (Hamblin, 2018). Moreover, its cost is much lower than repetitive TMS, electroconvulsive therapy (ECT), and vagus nerve stimulation (VNS), and there is no safety risk of selfadministration at home (Caldieraro and Cassano, 2019). Therefore, tPBM may gradually become a new alternative treatment method.

tPBM is a general type of photobiomodulation in which light penetrates the skull and enters the brain matter to provide effects (Salehpour et al., 2018). In this process, light passes through a series of layers, including scalp, periosteum, cranium and meninges, and causes neurobiological changes in turn (Chen et al., 2013; Rojas and Gonzalez-Lima, 2013). It is a potential neurorehabilitation treatment method in the field of brain related diseases (Jahan et al., 2019).

With the rapid development of tPBM in the field of cognitive neuroscience in recent years, we believe it is necessary to sort out and summarize the literature in this field. Therefore, this systematic review evaluates the changes in cognitive function of TBI patients after tPBM intervention and explores whether tPBM can repair the cognitive function of TBI populations by reviewing the use of cognitive testing methods in existing studies. Specifically, in this regard, we will try to determine the cognitive functions that can be improved. In addition, we will extract relevant treatment parameters, hoping to extract an optimal treatment plan that utilizes tPBM intervention to promote the cognitive rehabilitation of TBI patients. We will also identify existing problems based on the current research status and provide suggestions for future research.

## 2 Methods

The present systematic review was carried out following the PRISMA Statement for reporting systematic reviews (Moher et al., 2009, 2014).

### 2.1 Search strategy

We searched Web of Science, Cochrane, Scopus, EMBASE, PubMed and PsycInfo, using the following keywords: (“photobiomodulation” OR “low-level light therapy” OR “low-level laser therapy” OR “infrared light therapy” OR “infrared laser therapy” OR “transcranial laser” OR “transcranial light-emitting diode”) AND (“Traumatic brain injury” OR “Brain injury” OR “Head injury” OR “concussion”) AND (“cognition” OR “cognitive functioning”). The search cutoff date was 31 October 2023.

### 2.2 Screening procedure

The articles’ titles and abstracts were screened independently by two authors using inclusion and exclusion criteria. The inclusion criteria were: (1) evaluation of tPBM intervention; (2) traumatic brain injury population; (3) employed neuropsychological test or experimental paradigm in measuring participants’ cognitive functions. Exclusion criteria were: (1) reviews, conference articles, meeting abstracts, research poster; (2) guideline article, study protocol, expert opinions, editorials or commentaries; (3) animal studies; (4) lack of full text; and (5) lack of cognitive outcome measures. There were no restrictions based on language or publication date. In case of any disagreement, a third experienced reviewer was called upon to decide on the inclusion of a contested article (Sharma et al., 2020).

### 2.3 Quality assessment

Two reviewers used a tool developed by the Johanna Briggs Institute (JBI) to evaluate the quality of each included article. Each question has 4 potential answers to choose from, namely: “yes,” “no,” “clear” or “not applicable.” If there are differences, they will have a discussion to resolve or ask the third party for assistance in judging. JBI uses separate evaluation tools to evaluate the quality of each type of study (randomized control trial, cohort, case-study, qualitative investigation, etc.). The quality score for each study is the percentage of “yes” (low risk of bias) marks over the total number of criteria. A study was categorized as high quality, moderate quality, and low quality if the score is above 80%, between 50 and 80%, and below 50%, respectively (Lee et al., 2023).

## 3 Results

### 3.1 Sample

**Figure 1:**
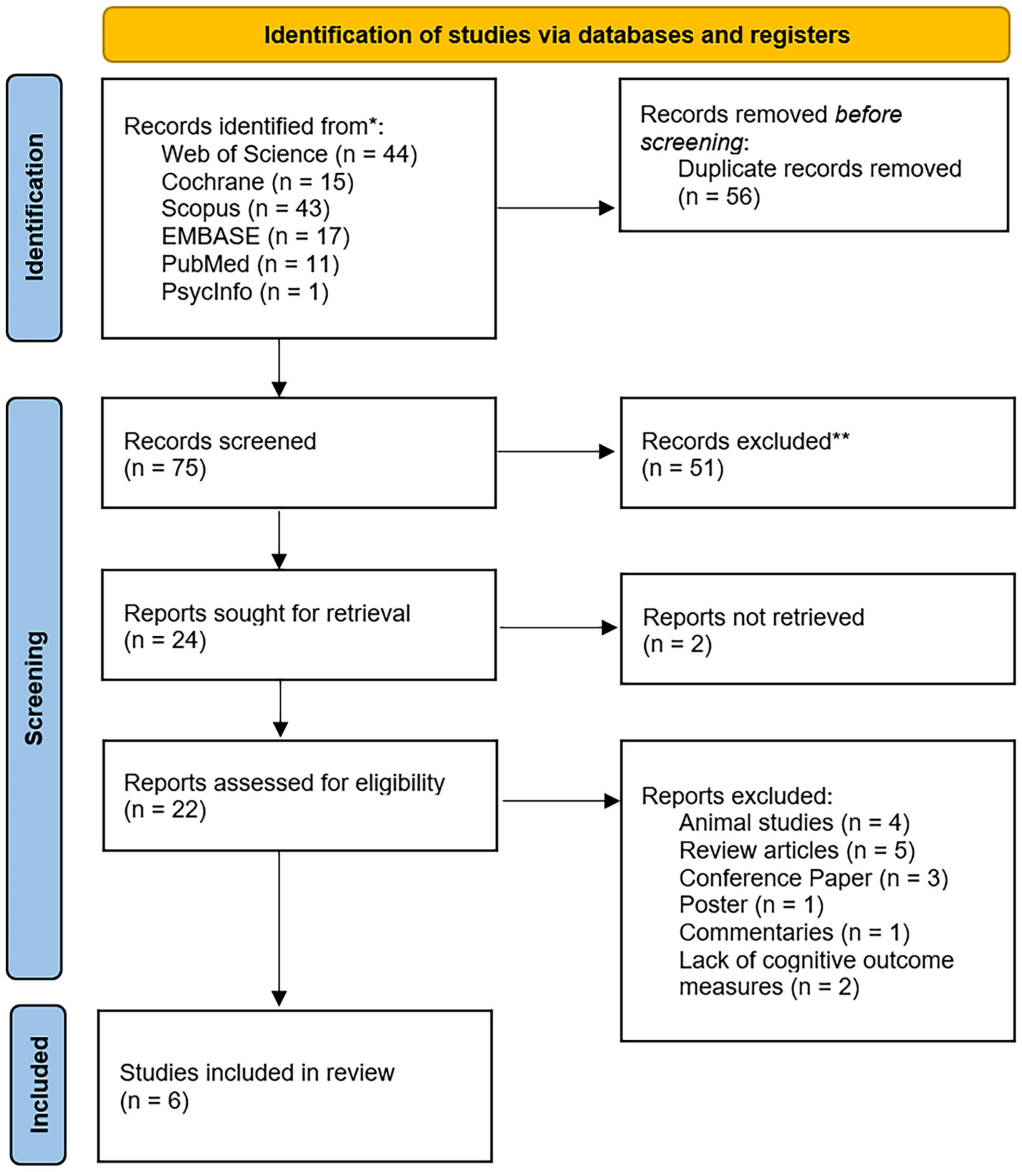
PRISMA flow diagram.

The initial search result consisted of 131 articles (Figure 1). The utilization of “Web of Science” resulted in 44 papers, “PubMed” in 11 articles, “Embase” in 17 articles, “Cochrane” in 15 articles, “Scopus” in 43 articles, “PsycInfo” resulted in 1 article, and “CINAHL” resulted in 0 articles. Irrelevant studies, review articles, conference papers, prospective articles, animal studies, or books were excluded. Six studies were included in this review. The six articles included two case reports (Naeser et al., 2011; Chao et al., 2020) and four case series (Naeser et al., 2014, 2023; Carneiro et al., 2019; Hipskind et al., 2019). There was a total of 40 TBI patients, including 11 males and 29 females, with an age range of 23–74 years. All the included patients were chronic TBI patients, four of whom were active or retired athletes.

### 3.2 Irradiation and light dose parameters

Currently, there are many tPBM devices used to treat different human brain diseases. Lasers and light emitting diodes (LEDs) have frequently been used for the head, but over time, LED arrays have increasingly become the most common method to deliver tPBM to the head. The six studies we included all used LED, not laser light. The wavelengths used across the studies were inconsistent. The red wavelengths ranged from 639 to 660 nm, and the near-infrared wavelengths, from 810 to 870 nm. The shortest wavelength was 629 nm (Hipskind et al., 2019) and the longest, 870 nm (Naeser et al., 2011, 2014).

In terms of the irradiation time per LED placement location on the scalp, per treatment, the most widely used time was 20 min (*n* = 3) (Hipskind et al., 2019; Chao et al., 2020; Naeser et al., 2023). One study used about 30 min (Carneiro et al., 2019). One study only used about 5 min for the LED placements for the first few treatments, and then gradually increased to about 10 min per placement (Naeser et al., 2011). One study always used 10 min per placement (Naeser et al., 2014). The most common treatment schedule was three treatments per week (Naeser et al., 2014, 2023; Hipskind et al., 2019; Chao et al., 2020), waiting one day in between, before the next treatment (e.g., Monday, Wednesday, Friday). With regards to the duration of a treatment series, the most common was 6 weeks (*n* = 4) (Naeser et al., 2014, 2023; Carneiro et al., 2019; Hipskind et al., 2019), and the longest was 9 months (*n* = 1) (Naeser et al., 2011). Another treatment series lasted for 8 weeks (Chao et al., 2020).

The irradiance (power density, mW/cm^2^) exhibited inconsistency across the studies. A power density of 22.2 mW/cm^2^ was the most frequently used (Naeser et al., 2011, 2014, 2023). Next, power densities of 75 mW/cm^2^ and 100 mW/cm^2^ were used (Chao et al., 2020; Naeser et al., 2023). The Hipskind et al. (2019) study used a pulsed power density of 6.4 mW/cm^2^. The minimum irradiance was 2.075 mW/cm^2^ (Carneiro et al., 2019). In addition, fluency (energy density, J/cm^2^) showed large variation. The most common energy density was 26 J/cm^2^ (Naeser et al., 2011, 2014, 2023). Other energy densities were 60 J/cm^2^, 45 J/cm^2^, and 15 J/cm^2^ (Chao et al., 2020; Naeser et al., 2023). The Hipskind et al. (2019) study used a pulsed energy density of 7.7 J/cm^2^. The range of total energy delivered was large, ranging from 4,536 J (Naeser et al., 2023) to 271,107.18 J (Naeser et al., 2011). Four studies used continuous wave (Naeser et al., 2011, 2014, 2023; Carneiro et al., 2019), and two studies used pulsed wave (Hipskind et al., 2019; Chao et al., 2020) (Table 1).

**Table 1:** Assessment used and treatment outcome of tPBM studies.

Source	Subject (n)	Light-emitting device	Wavelength (nm)	Irradiance (mW/cm^2^)	Fluency (J/cm^2^)	Total energy delivered	Wave type	Treatment location	Treatment period	Pre- and post-tests	Main outcome
Naeser et al. (2011)	Chronic TBI (2)	LED	633; 870	22.2	9.324 (7 min)–13.32 (10 min)	271107.18	Continuous	3 (Entire head, forehead, frontal, parietal, and temporoparietal)	1st week: 7 min; 2nd week: 8 min; 3rd week: 9 min thereafter: 10 min/9 months	Stroop; WMS-R	Improved executive function (inhibition) and memory, reduction in PTSD.
Chao et al. (2020)	TBI (1)	LED	810	Posterior:100; anterior:75; intranasal:25	Posterior:60; anterior:45; intranasal:15	6,720	Pulsed wave 10 Hz, 40 Hz	5 (DMN; intranasal)	20 min/28/ Tx.’s 8 weeks	CVLT-II; TMT; VFT; CWIT; WAIS-III	Improved verbal learning and memory, executive function, attention, verbal fluency and processing speed.
Naeser et al. (2023)	Chronic TBI (4)	LED	Protoc. A: 633, 870 Protoc. B: 810, 633 Protoc. C: 660,850	Protoc. A: 22.2; Protoc. B: 75, 100, 25; 8 Protoc. C: 41; 35	Protoc. A&C: 26 Protoc. B: 45, 60, 15; 12	Protoc. A: 10,800 Protoc. B: 4,536 Protoc. C: 29,259	Protoc. A&C: Continuous Protoc. B: 40	Protocol A&C: Entire head Protocol B: 810, DMN, and 630, intranasal	22–40 min/ 18/6 weeks	Stroop; TMT; CVLT-II; CPT; CWIT; BVMT-R; COWAT/FAS	Improved executive function; attention; verbal learning and memory; visuospatial memory; verbal fluency.
Carneiro et al. (2019)	Chronic TBI (10)	LED	630	2.075	3.74	26,892	Continuous	1 (Entire head)	30 min/18/6 weeks	Stroop; TMT; DST; RCFT; RAVLT; VFT	Improve in visual and verbal episodic memory, planning, and processing speed.
Hipskind et al. (2019)	Chronic TBI (12)	LED	629, 850	6.4	7.7	71,892	Pulsed wave 73 Hz; 587 Hz; 1,175 Hz	1 (Entire head)	20 min/18/6 weeks	CVLT-II; WAIS-IV; TMT-B; DVT	6 of 15 neuropsychological tests, improved verbal learning; memory; concentration; processing speed.
Naeser et al. (2014)	Chronic TBI (11)	LED	630, 870	22.2	26.64	64,800	Continuous	2 sets of 6 LED Cluster Heads (Entire head, frontal, parietal, temporal areas)	20 min/18/6 weeks	TMT; Stroop; CVLT-II; COWAT/FAS Test	Improved executive function and verbal learning and memory.

WMS-R, the Wechsler Memory Scale – Revised; CVLT-II, California Verbal Learning Test II; CWIT, Color Word Interference test; TMT, Trail Making Test; BVMT, Brief Visuospatial Memory Test; CPT, Continuous Performance Test; WAIS-III, Digit Span subtest of the Wechsler Adult Intelligence Scale-III; VFT, Verbal Fluency Test; COWAT/FAS Test, Controlled Oral Word Association Test; DST, Digit Symbol Test; RAVLT, Rey Auditory Verbal Learning Test; RCFT, Rey Complex Figure Test; WAIS-IV, Wechsler Adult Intelligence Scale IV; DVT, Digit Vigilance Test; PTSD, post-traumatic stress disorder.

### 3.3 Treatment location and mode of application

There were differences in the specific treatment locations in the different studies. Most studies primarily used the entire head of the subject, including frontal, parietal and temporal lobes as the treatment locations (*n* = 5) (Naeser et al., 2011, 2014, 2023; Carneiro et al., 2019; Hipskind et al., 2019). One study used photobiomodulation therapy for only the nasal cavity, in addition to the standard cortical nodes for the Default Mode Network (Chao et al., 2020). One study also treated the Default Mode Network starting around 2 months after the first series of whole-head tPBM treatments had been completed (Naeser et al., 2023).

### 3.4 Pre- and post-testing

Cognitive testing included multiple components, and the tests used were diverse. The Trail Making Test was the most frequently used (*n* = 5) (Naeser et al., 2014, 2023; Carneiro et al., 2019; Hipskind et al., 2019; Chao et al., 2020). Four studies used the Stroop (Naeser et al., 2011, 2014, 2023; Carneiro et al., 2019) and the California Verbal Learning Test II (Naeser et al., 2014, 2023; Hipskind et al., 2019; Chao et al., 2020). Two studies used the Controlled Oral Word Association Test (COWAT)/FAS Test (Naeser et al., 2014, 2023) and Color Word Interference Test (Chao et al., 2020; Naeser et al., 2023), and the Wechsler Adult Intelligence Scale (Hipskind et al., 2019; Chao et al., 2020). Other tests used among the six studies reviewed included the Rey Auditory Verbal Learning Test; the Complex Rey Figure; verbal and category fluency; the Wechsler Memory Scale, the Digit Vigilance Test, Continuous Performance Test, Brief Visuospatial Memory Test and the Symbol Digit Test.

### 3.5 Intervention effects

Thus far, most of the clinical studies have been conducted on patients with chronic TBI. People who have had head injuries can suffer from a variety of long-term symptoms, including cognitive problems with impaired executive function, difficulty in concentration, and poor memory. Each study reviewed here, examined the intervention effect of tPBM on cognitive function. It was found that these studies examined multiple aspects of cognitive function. All researchers pointed out that the memory of subjects was enhanced after tPBM intervention (*n* = 6). In addition, 5 out of 6 studies reported improvements in the subjects’ executive function (Naeser et al., 2011, 2014, 2023; Carneiro et al., 2019; Chao et al., 2020). It should be noted that the aspect of improved executive function referred to in these studies was mainly improved inhibition, as tested with the Stroop test. Improved cognitive processing speed in TBI patients treated with tPBM has also received significant attention. Three studies reported that tPBM accelerated cognitive processing speed (Carneiro et al., 2019; Hipskind et al., 2019; Chao et al., 2020). Four studies reported that tPBM intervention improved verbal learning and memory (Naeser et al., 2014, 2023; Hipskind et al., 2019; Chao et al., 2020). Four studies observed improvement in executive function/inhibition on the Stroop, including later home treatment (Naeser et al., 2011, 2014, 2023; Chao et al., 2020). Three studies observed positive changes in the subjects’ attention and concentration after the tPBM intervention (Hipskind et al., 2019; Chao et al., 2020; Naeser et al., 2023). Two studies reported that tPBM intervention improved verbal fluency (Chao et al., 2020; Naeser et al., 2023). In addition, two case series reported that tPBM intervention had a significant impact on visual and verbal episodic memory and visuospatial memory (Carneiro et al., 2019; Naeser et al., 2023).

It should be emphasized that these early clinical studies using LEDs to treat TBI were neither controlled nor randomized. This included results from two case reports (Naeser et al., 2011; Chao et al., 2020), and four case series (Naeser et al., 2014, 2023; Carneiro et al., 2019; Hipskind et al., 2019). We did not retrieve any eligible randomized, controlled trial (RCT) studies. Thus, the results in the six studies reviewed could include a possible placebo effect. This is unknown. Future studies need to include randomized, controlled trials.

### 3.6 Quality assessment

**Figure 2:**
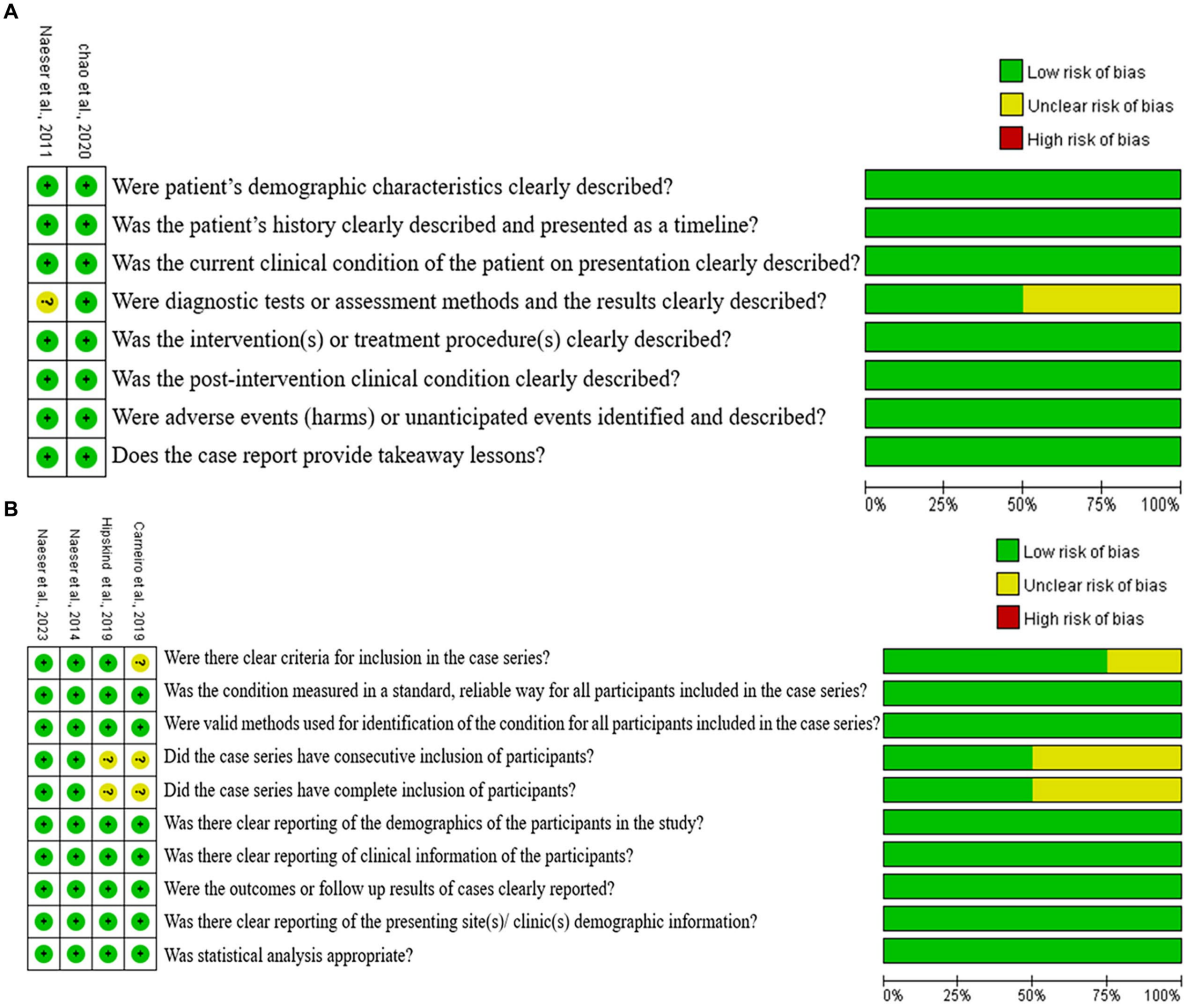
Cochran risk of bias tool. Each risk of bias item for each included study and presented as percentages across all included studies. **(A)** Case report, **(B)** case series.

Figure 2 shows the results for the overall quality of the two case-report studies, and the four case-series studies. For the six articles, five studies (Naeser et al., 2011, 2014, 2023; Hipskind et al., 2019; Chao et al., 2020) (83.3%) were evaluated as high quality (e.g., low bias risk), while the remaining one study (Carneiro et al., 2019) (16.7%) was rated as medium quality. The methodological quality of most of the included studies was considered high.

## 4 Discussion

The purpose of this study was to systematically evaluate the effectiveness of tPBM to reduce cognitive impairments in patients with TBI. In addition, the different tPBM treatment parameters used in six different studies were reviewed. The cognitive functions evaluated included executive function (inhibition), verbal learning and memory, attention, and concentration. Overall, our results showed that tPBM can promote positive changes in cognitive function in persons with TBI. This suggests that tPBM may be a promising technology for treating TBI patients with cognitive impairment.

Our results are basically consistent with previous meta-analysis results from different subject populations. For example, Salehpour et al.’s meta-analysis found that tPBM can have beneficial effects on memory, attention, and executive function in healthy individuals (Salehpour et al., 2019). Furthermore, meta-analysis studies that focused on patients with MCI, dementia, stroke, Parkinson’s and Alzheimer’s disease, also found that tPBM was beneficial to promote recovery of cognitive function (Spera et al., 2021; Cheung et al., 2023; Kuo and Kim, 2023; McGee et al., 2023).

However, no large-scale, controlled clinical trials have been completed that used tPBM to treat the cognitive function of chronic TBI. Thus, the application of tPBM to improve cognition after TBI has not been widely accepted. At present, only one RCT study was found that used tPBM with TBI (Longo et al., 2020). The subjects were TBI patients in the acute, early subacute, and late subacute stage and their cognitive function was not evaluated before and after the intervention. In various studies, however, neuroimaging measures have provided objective support for cognitive improvements following a series of tPBM treatments (Hipskind et al., 2019; Chao et al., 2020; Naeser et al., 2023).

Chao et al. (2020), examined a 23-year-old professional ice hockey player who had suffered 6 concussions in 5.5 years, using brain magnetic resonance imaging (MRI) before and after a series of tPBM treatments. They found that after these treatments, the volume of total cortical GM, subcortical GM, and thalamus in this athlete increased. In addition, these researchers observed that after the 8 weeks of tPBM treatments, there was a less scattered presence of functional connectivity (FC) among several cortical areas. There was a better-defined FC, for example, between the anterior cingulate cortex (ACC) and the anterior insula—both are cortical nodes of the Salience Network (SN) which is important for attention. Initially after head injury, undamaged parts of the brain may attempt to take over the function of injured areas (Stevens et al., 2012; Iraji et al., 2015). After tPBM intervention, there appears to be less demand for compensatory processes (Chao et al., 2020).

Hipskind et al. conducted single photon emission computed tomography (SPECT) brain scans on TBI participants before and after intervention to quantitatively measure regional CBF (rCBF) in 138 regions of interest. After intervention, 8 out of 12 participants showed a significant increase in rCBF (Hipskind et al., 2019).

Carneiro et al. compared the cerebral hemodynamic conditions of TBI patients before and after tPBM intervention through transcranial Doppler (TD). It was found that tPBM promoted increased rCBF, mainly in left peak systolic velocity (PSV), thereby increasing cerebral oxygenation, and in turn, improving cerebral function (Carneiro et al., 2019).

Naeser et al. (2023) investigated the brain changes in TBI participants before and after a series of tPBM treatments using MRI. Resting-state functional-connectivity MRI (rs-fcMRI) showed an increase in FC within SN, and within the central executive network (CEN) following a 6-week tPBM treatment series. Magnetic resonance spectroscopy (MRS) analysis showed an increase in the metabolite, n-acetyl-aspartate (NAA), in ACC. Further analysis revealed that rs-fcMRI showed significant correlations with increases in the SN FC with executive function, attention, post-traumatic stress disorder (PTSD), pain, and improved sleep. The increase in CEN FC was related to improved verbal learning and memory, as well as depression. The increase in NAA in the ACC was related to reduced pain and PTSD. These changes on rs-fcMRI, and on MRS support the beneficial cognitive effects of tPBM (Naeser et al., 2023).

Although the six articles included in this review observed similar beneficial findings, there were differences in the tPBM LED parameters used. Thus, at this time, there is no consensus on the optimal treatment parameters for clinical application of tPBM for chronic TBI.

Currently, there are mainly two types of light sources used in tPBM: lasers and LEDs. tPBM is a radiant energy that utilizes wavelengths ranging from red to near-infrared, with high penetration to small tissue areas (Gutiérrez-Menéndez et al., 2020). PBM uses laser or LED light to regulate biological functions and achieve specific therapeutic effects in a noninvasive manner. Some studies suggest that a large amount of laser energy will cause tissue heating, which may increase the risk of damage (Rojas and Gonzalez-Lima, 2013). The use of LEDs is relatively safe and less expensive than lasers. They may illuminate large areas of tissue at the same time. In addition, LEDs do not need to consider the optical safety issues of lasers (Montazeri et al., 2021). Flexible LED devices can be embedded into helmets for tPBM application (Salehpour et al., 2019). These advantages have encouraged increasingly more researchers to use LEDs instead of lasers (Berman et al., 2017; Salehpour and Rasta, 2017). Perhaps due to the above reasons, all six studies included in this review adopted LED light.

Wavelength is one of the important parameters to consider for tPBM treatment. The wavelength should be selected within a reasonable range, to provide better absorption and sufficient tissue penetration to reach the mitochondria and cytochrome c oxidase (CCO) in the targeted cells (Carroll, 2019). This range is between 600 and 1,200 nanometers, except between 700 and 780 nm where there is a potential for reduced efficacy (de Freitas and Hamblin, 2016; Henderson and Morries, 2017; Carroll, 2019). Similar to this view, studies have shown that light in the spectral bands of 650–680 and 800–870 nm match the absorption of relatively oxidized CCO, while the band at 750–770 nm matches reduced CCO (Karu et al., 2005). On this basis, regarding the TBI studies reviewed in this paper—although the wavelengths were inconsistent, they were primarily 630–660 nm and 810–870 nm. This range was associated with beneficial effects on cognition in the chronic TBI cases treated.

In research on TBI, most studies have set the irradiance at 10–70 mW/cm^2^, with 22.2 mW/cm^2^ as the most commonly used. The irradiance depends on the number of watts of the device, and the size of the area in cm^2^ that is emitting the photons from that device. In order to ensure patients’ safety, the values used in the medical field are usually low (Gavish and Houreld, 2018). 10 to 70 mW/cm^2^ is the typical LED irradiance used in studies (Cassano et al., 2015; Salgado et al., 2015). The irradiance parameters used in intranasal applications are lower than those used in transcranial applications (Gutiérrez-Menéndez et al., 2020).

Concerning energy density, previous studies have pointed out that the most commonly used doses for treating neurological disorders are between 10 and 30 J/cm^2^, and for treating psychological disorders, between 12 and 84 J/cm^2^. The recommended range for healthy subjects is between 15 and 60 J/cm^2^ (Karu et al., 2005; Cassano et al., 2015). In this study, the energy density parameters used by the researchers were different, primarily ranging from 12 to 30 J/cm^2^ per LED placement (Naeser et al., 2011, 2014, 2023; Chao et al., 2020), with a few reaching 60 J/cm^2^ (Chao et al., 2020; Naeser et al., 2023). We also found significant differences in total energy among different studies (e.g., ranging from around 4,536 to 271,107.18 J) (Naeser et al., 2011, 2023). Due to the beneficial intervention effects achieved in all six of the included studies, we speculate that the total energy dose for TBI patients should not be too low. However, we also suggest that it may not be necessary to provide too high doses of total energy in order to promote cognitive recovery in TBI patients.

Regarding the specific areas on the head to treat in TBI, because the forehead has little hair, it has been used frequently. The frontal pole areas are just behind the forehead. In addition, one can likely deliver photons to the midline, mesial prefrontal cortex area. The mesial prefrontal cortex area is an important node in the Default Mode Network. Thus, the forehead was a commonly selected area for tPBM in the studies reviewed (Naeser et al., 2011, 2014, 2023; Carneiro et al., 2019; Hipskind et al., 2019; Chao et al., 2020). In addition, the parietal (and temporal lobes) have been treated in studies with tPBM (Naeser et al., 2011, 2014, 2023; Carneiro et al., 2019; Hipskind et al., 2019; Chao et al., 2020). Some studies applied the tPBM treatments to the entire head (Naeser et al., 2014, 2023; Carneiro et al., 2019; Hipskind et al., 2019). Some studies also delivered photons to the nasal cavity (Chao et al., 2020; Naeser et al., 2023). tPBM delivered to anatomical sites outside the scalp (such as the nasal cavity) can allow photons to be absorbed by circulating blood which can then benefit the brain (Lee et al., 2023).

In regards to the irradiation time during a treatment session, many studies chose a treatment time of 20 min per LED placement (Hipskind et al., 2019; Chao et al., 2020; Naeser et al., 2023). Lapchak et al. suggested that patients should receive multiple tPBM treatments during a tPBM treatment series for best results (Lapchak, 2012). Many studies with TBI patients applied a total of 18 tPBM treatments, three times per week (with a 48-h break, between treatments), over a time-period of 6 weeks (Naeser et al., 2014, 2023; Carneiro et al., 2019; Hipskind et al., 2019). In addition, we have analyzed safety issues during the treatment process. In the six studies included, four studies clearly reported no side effects or adverse events (Naeser et al., 2011, 2014, 2023; Hipskind et al., 2019). One study did not mention the safety issues (Carneiro et al., 2019). Chao et al. pointed out in the case report that the subject had mild headaches after one week of receiving tPBM treatment with the Vielight Neuro Gamma headframe device, pulsed at 40 Hz. After 10 days of adjusting to a different headframe device, the Vielight Neuro Alpha device, pulsed at 10 Hz, the headaches were no longer experienced (Chao et al., 2020). The 40 Hz pulse rate is considered to be more stimulating, whereas the 10 Hz pulse rate is more relaxing (Zomorrodi et al., 2019). Overall, the treatment of tPBM is relatively safe and has not caused any significant safety issues.

In summary, through a review of the relevant tPBM literature to treat chronic TBI, it appears that this treatment method can promote improved cognition. There were differences, however, in details of the tPBM parameters used in each study. At this time, there is no consensus. Also, the early research in this field, which includes case reports and case series, has not had an opportunity to enroll sham-control groups. Thus, it is not clear if the improvement in cognition for these chronic TBI cases was entirely associated with the tPBM treatments.

Although the current literature supports the use of tPBM as a promising treatment to improve cognition in chronic TBI patients, in order to have sufficient evidence, large, randomized, controlled clinical trials are necessary (Lee et al., 2023). Future tPBM research with chronic TBI cases could offer optimal treatment parameters. Also to date, most research with TBI has been performed with chronic TBI in humans; the research with acute TBI has mainly only been conducted on animal models (Stevens et al., 2023). At present, it is unknown if tPBM is safe and effective for use with acute TBI patients. Since tPBM has been shown to promote increase in rCBF, it would be contraindicated in cases with active bleeding—e.g., acute brain hemorrhage in humans, until bleeding has completely stopped. Finally, it is likely with the development of, and more widespread use of better brain imaging techniques—e.g., rs-fcMRI, MRS, SPECT and PET scans, the mechanisms and effects of tPBM to improve cognitive function in TBI will become better known.

With the increasing incidence of TBI worldwide, cognitive rehabilitation methods for acute and chronic TBI patients requires attention. Treatments that include tPBM offer promise, at this time but more sham-controlled clinical research studies are needed.

## 5 Conclusion

In conclusion, this systematic review of two case reports, and four case series studies using tPBM with chronic TBI patients suggests that tPBM can be used to improve cognition. tPBM is non-invasive, and safe as used in these six tPBM studies. Cognitive function was improved in the areas of executive function (inhibition), processing speed, attention, concentration, verbal learning and memory, and verbal fluency. tPBM was reported to increase the volume of total cortical GM, subcortical GM, and thalamus, improve CBF, FC, and cerebral oxygenation. These may be some key mechanisms associated with improved cognition in the chronic TBI patients studied. The specific tPBM treatment parameters varied, and future studies should refine them for optimal effects. Given these encouraging findings in the existing literature, it is recommended that high-quality, large, randomized controlled clinical trials be conducted with acute as well as chronic TBI patients.

## Data availability statement

The original contributions presented in the study are included in the article/supplementary material, further inquiries can be directed to the corresponding author.

## Author contributions

JZ: Conceptualization, Data curation, Investigation, Methodology, Writing – original draft, Writing – review & editing. CW: Data curation, Methodology, Supervision, Writing – original draft, Writing – review & editing. YC: Data curation, Methodology, Supervision, Visualization, Writing – review & editing. DL: Data curation, Methodology, Supervision, Writing – review & editing. QW: Supervision, Validation, Writing – review & editing.

## Glossary

tPBM: transcranial photobiomodulation
TBI: traumatic brain injury
GM: cortical gray matter
CBF: cerebral blood flow
TES: transcranial electrical stimulation
TMS: transcranial magnetic stimulation
ECT: electroconvulsive therapy
VNS: vagus nerve stimulation
LEDs: light emitting diodes
COWAT: Controlled Oral Word Association Test
MRI: magnetic resonance imaging
ACC: anterior cingulate cortex
SPECT: single photon emission computed tomography
TD: transcranial Doppler
PSV: peak systolic velocity
rs-fcMRI: resting state functional connectivity MRI
SN: salience network
CEN: central executive network
MRS: magnetic resonance spectroscopy
NAA: n-acetyl-aspartate
PTSD: post-traumatic stress disorder
CCO: cytochrome c oxidase
